# Shotgun metagenomic data reveals significant abundance but low diversity of “*Candidatus* Scalindua” marine anammox bacteria in the Arabian Sea oxygen minimum zone

**DOI:** 10.3389/fmicb.2014.00031

**Published:** 2014-02-05

**Authors:** Laura Villanueva, Daan R. Speth, Theo van Alen, Alexander Hoischen, Mike S. M. Jetten

**Affiliations:** ^1^Department of Marine Organic Biogeochemistry, Royal Netherlands Institute for Sea ResearchDen Burg, Netherlands; ^2^Department of Microbiology, IWWR, Radboud University NijmegenNijmegen, Netherlands; ^3^Department of Human Genetics, UMC St. RadboudNijmegen, Netherlands; ^4^Department of Biotechnology, Delft University of TechnologyDelft, Netherlands

**Keywords:** nitrogen cycle, anaerobic ammonium oxidation, metagenome, hydrazine, marine anammox

## Abstract

Anaerobic ammonium oxidizing (anammox) bacteria are responsible for a significant portion of the loss of fixed nitrogen from the oceans, making them important players in the global nitrogen cycle. To date, marine anammox bacteria found in both water columns and sediments worldwide belong almost exclusively to “*Candidatus* Scalindua” species. Recently the genome assembly of a marine anammox enrichment culture dominated by “*Candidatus* Scalindua profunda” became available and can now be used as a template to study metagenome data obtained from various oxygen minimum zones (OMZs). Here, we sequenced genomic DNA from suspended particulate matter recovered at the upper (170 m deep) and center (600 m) area of the OMZ in the Arabian Sea by SOLiD and Ion Torrent technology. The genome of “*Candidatus* Scalindua profunda” served as a template to collect reads. Based on the mapped reads marine anammox Abundance was estimated to be at least 0.4% in the upper and 1.7% in the center area. Single nucleotide variation (SNV) analysis was performed to assess diversity of the “*Candidatus* Scalindua” populations. Most highly covered were the two diagnostic anammox genes hydrazine synthase (scal_01318c, *hzs*A) and hydrazine dehydrogenase (scal_03295, *hdh*), while other genes involved in anammox metabolism (*nar*GH, *nir*S, *amt*B, *foc*A, and ACS) had a lower coverage but could still be assembled and analyzed. The results show that “*Candidatus* Scalindua” is abundantly present in the Arabian Sea OMZ, but that the diversity within the ecosystem is relatively low.

## Introduction

Anaerobic ammonium oxidation (anammox) is mediated by a specialized group of bacteria that have many unique properties including the synthesis of ladderane lipids (Damsté et al., 2002), the presence of a prokaryotic cell organelle (van Niftrik and Jetten, 2012), and a metabolism using reactive intermediates hydrazine and nitric oxide (Kartal et al., 2011, 2013). Most anammox bacterial cultures have been enriched from wastewater treatment environments, and therefore much of anammox research is directed toward application of these bacteria in sustainable man-made treatment systems (Kartal et al., 2010). In addition, it has become clear that anammox bacteria contribute significantly to the loss of fixed nitrogen from both marine and terrestrial ecosystems (Kuypers et al., 2003, 2005; Zhu et al., 2013).

To date, at least five genera of anammox bacteria have been enriched and described, and these form a monophyletic order of the *Brocadiales* that branches deeply in the phylum Planctomycetes (Jetten et al., 2010). The deepest branching anammox genus, “*Candidatus* Scalindua” (hereafter referred to as Scalindua), is found in all marine environments investigated worldwide (Schmid et al., 2007; van de Vossenberg et al., 2008; Woebken et al., 2008; Hong et al., 2011; Dang et al., 2013). Scalindua bacteria may interact with both thaumarchaeotal and bacterial ammonium-oxidizing microbes under oxygen limitation (Lam et al., 2009; Yan et al., 2012) for their nitrite supply. For most of the anammox genera, draft genome assemblies are available (Strous et al., 2006; Gori et al., 2011; Hira et al., 2012; Hu et al., 2012; Speth et al., 2012; van de Vossenberg et al., 2012).

First studies on anammox bacteria diversity based on 16S rRNA gene sequences in suboxic waters, as well as marine and freshwater sediments, concluded that all environmental sequences were closely related to the “*Candidatus* Scalindua” genus and that the diversity was generally low in comparison with other systems, such as wastewater treatment plants (Woebken et al., 2007; Hu et al., 2010). However, another study by Woebken et al. (2008) revealed a significant microdiversity within the Scalindua genus by sequence analyses of 16S rRNA genes and the 16S–23S rRNA internal transcribed spacer. So far 16S rRNA, hydrazine synthase (*hzs*A), hydrazine dehydrogenase (*hdh*), and *nir*S genes have been used to estimate the diversity and abundance of anammox bacteria (Schmid et al., 2005, 2008; Li et al., 2011; Harhangi et al., 2012). The primers used for amplification of anammox 16S rRNA genes are hampered by their lack of specificity (Schmid et al., 2005; Woebken et al., 2008). In the most extreme case only 1 in 480 clones analyzed could be attributed to anammox in a sample from the Black Sea (Woebken et al., 2008). *Nir*S genes seem only be present in the genera Scalindua and Kuenenia genome assemblies, while Planctomycete KSU-1, Jettenia and *Brocadia* seem to employ a *nir*K type of nitrite reductase, limiting the use of *nir*S as biomarker for the detection of anammox bacteria (Gori et al., 2011; Hira et al., 2012; Hu et al., 2012). Hydrazine dehydrogenase genes are present in multiple divergent copies in each anammox genome assembly making their use as phylomarkers at least cumbersome (Schmid et al., 2008). Recently primers for the amplification of hydrazine synthase genes became available (Harhangi et al., 2012), and were shown to be quite specific and sensitive. Nevertheless, PCR primers are never better than the data they are based on and thus primer independent methods to study anammox diversity are highly desirable. Here we used shotgun metagenome data obtained by Next Generation Sequencing (NGS) of DNA from two depths in the Arabian Sea OMZ to retrieve non primer-biased information on the biodiversity of marine anammox bacteria in these ecosystems. The oxygen minimum zone (OMZ) of the Arabian Sea represents a globally important site for oceanic nitrogen loss (Lam and Kuypers, 2011; Pitcher et al., 2011). Its vertical distribution extends from approximately 150–2000 meters below sea level with very low oxygen concentrations, making it one of the most expansive and intense OMZ in the global ocean. Loss of fixed nitrogen from the Arabian Sea OMZ may occur via both denitrification and anammox (Ward et al., 2009; Jensen et al., 2011) although the contribution of each process may vary with season and location.

Earlier work using shotgun metagenomic and metatranscriptomic data has shown that anammox bacteria are abundant and active at the core of the Eastern Tropical South Pacific OMZ off Chile OMZ (Stewart et al., 2012), but likely underestimated anammox abundance due to the absence of a closely related reference sequence. Here, we used the recently available genomic template of “*Candidatus* Scalindua profunda” (van de Vossenberg et al., 2012) to assess anammox abundance and diversity through 16S rRNA gene reads and the marker genes hydrazine synthase and hydrazine dehydrogenase.

## Materials and methods

### Sampling and sample preparation

Arabian Sea depth profiles are described in (Pitcher et al., 2011). Large-volumes of seawater (200–1700 L) were filtered through 142-mm diameter 0.2-μm polycarbonate filters (Millipore, Billerica, MA). Filters were cut into fragments prior to extraction. Cells were lysed by bead-beating with 1.5 g of sterile 0.1 mm zirconium beads (Biospec, Bartlesville, OK) in a extraction buffer containing 10 mM Tris-HCl pH 8, 25 mM Na_2_ EDTA pH 8, 1% (v/v) sodium dodecyl sulfate (SDS), 100 mM NaCl, and molecular biology grade water. Samples were incubated at 70°C for 30 min and then extracted with phenol-chloroform (Sambrook et al., 1989). After extraction, DNA was precipitated using ice-cold ethanol, dried, and re-dissolved in 100 μl of 10 mM Tris-HCl, pH 8. Total nucleic acid concentrations were quantified spectrophotometrically (Nanodrop, Thermo Scientific, Wilmington, DE, USA) and checked by agarose gel electrophoresis for quality. Extracts were kept frozen at −80°C.

### Metagenome sequencing

#### SOLiD library preparation and sequencing

Aliquots of DNA from station PA2 (170 m) and PA5 (600 m) were prepared for SOLiD libraries following manufacturer's instructions (Life Technologies, Carlsbad, CA, USA). In essence 5 μg genomic DNA (gDNA) was used as input material, sheared to ~180 bp fragments, end-repaired, barcoded sequencing adaptors were ligated by blunt-end ligation. Finished libraries were analyzed by Bioanalyzer (Agilent, Santa Clara, CA, USA) and by Qubit (Life Technologies, Carlsbad, CA, USA) prior to use. Four libraries were pooled in equimolar amounts, and E80 ePCR was performed following manufacturer's instructions (Life Technologies, Carlsbad, CA, USA) with 0.7 pM final library concentration. Each E80 library pool consisting of 4 samples was finally sequenced on one SOLiD4 sequencing slide (Table A1). For PA2 one library was prepared while for PA5 two independent libraries were prepared to yield sufficient data, and run on a SOLiD0753. The 50 nt color coded reads were separated according to barcode and analyzed using the CLC genomics workbench (v4.9, CLCbio, Aarhus, Denmark) as described below.

#### Ion torrent library preparation and sequencing

All kits used in this section were obtained from Life technologies (Life technologies, Carlsbad, CA, USA). For both samples an identical library preparation was performed. Genomic DNA (extraction described above) was sheared for 7 min using the Ion Xpress™ Plus Fragment Library Kit following the manufacturer's instructions. Further library preparation was performed using the Ion Plus Fragment Library Kit following manufacturer's instructions. Size selection of the library was performed using an E-gel 2% agarose gel, resulting in a median fragment size of approximately 330 bp. Emulsion PCR was performed using the Onetouch 200 bp kit and sequencing was performed on an Ion Torrent PGM using the Ion PGM 200 bp sequencing kit and an Ion 318 chip.

#### Extraction and analysis of reads similar to “candidatus scalindua profunda”

The genome of “*Candidatus* Scalindua profunda” was used as a template to harvest reads using read mapping as implemented in the CLC genomics workbench. Matching reads (mismatch penalty 2, In/Del penalty 3) and at least 80% identity over 50% of the read length were extracted. Subsequently, the identity of the reads was confirmed with a BLASTx analysis against the “*Candidatus* Scalindua profunda” proteins, using an E-value cut-off of 1 for SOLiD reads and 1e^−4^ for IonTorrent reads (Stewart et al., 2012). To assess diversity of “*Candidatus* Scalindua profunda” in the samples a single nucleotide variation (SNV) analysis was performed on consensus sequences of hydrazine synthase (scal_01317, *hzs*A) and hydrazine dehydrogenase (scal_03295, *hdh*) genes using the CLC genomics workbench. Consensus sequences were generated by iterative mapping of the Ion Torrent PA5 reads as described previously (Dutilh et al., 2009), using the CLC genomics workbench read mapper with mismatch penalty 2, In/Del penalty 3 and at least 80% identity over 50% of the mapping reads.

For 16S rRNA gene phylogenetic analysis, reads matching the “*Candidatus* Scalindua profunda”16S rRNA gene with a positive score (mismatch penalty 2, In/Del penalty 3) and at least 90% identity over 50% of the read length were extracted. These parameters result in an overestimation, thus subsequent phylogenetic assignment is required to remove false positives.

### Phylogenetic analysis

The phylogenetic affiliation of the partial bacterial 16S rRNA gene sequences affiliated to the Planctomycetes group were compared to release 111 of the Silva SSU Ref database [http://www.arb-silva.de; (Quast et al., 2012)] using the ARB software package (Ludwig et al., 2004). The partial sequences generated in this study were added to the reference tree supplied by the Silva database using the ARB Parsimony tool. Putative and annotated *hzs*A gene sequences were translated to protein and aligned by Muscle (Edgar, 2004) in Mega5 software (Tamura et al., 2011) and edited manually. Phylogenetic reconstruction of putative hydrazine synthase proteins was performed by maximum likelihood in PhyML v3.0 (Guindon et al., 2010) using the LG model plus gamma distribution including estimated amino acid frequencies (LG + G + F) indicated by ProtTest 2.4 (Abascal et al., 2005). Branch support was calculated with the approximate likelihood ratio test (aLRT).

## Results and discussion

### Sampling sites and overview of sequencing results

The Arabian Sea is one of the most expansive and intense OMZ in the global ocean, and presence of anammox bacteria through activity tests and molecular surveys has been documented previously (Jensen et al., 2011; Pitcher et al., 2011) We used gDNA from suspended particulate matter obtained at 170 m (station PA2), at the upper limit of the OMZ, and at 600 m depth (station PA5), at the core of the OMZ, that was collected during a sampling campaign in January 2009 (for details see Pitcher et al., 2011). The gDNA from both stations was subjected to SOLiD and Ion Torrent sequencing (Table 1) yielding 7.8 and 8.6 gigabases of information for station PA2 and PA5 respectively. At the upper station PA2 between 0.4 and 0.9 percent of the reads matched to coding regions of the “*Candidatus* Scalindua profunda” genome (van de Vossenberg et al., 2012), while at the core OMZ station PA5 between 1.7 and 3.5 percent of the reads matched. The estimated percentage of *Scalindua sp.* based on the SOLiD reads is probably an overestimate, as BLASTx analysis on the matching reads revealed that about 28% of those reads did not give a hit to a “*Candidatus* Scalindua profunda” protein (Table A1) using an E-value of 1. The longer Ion Torrent reads gave a more robust estimate, as only 0.3% of the reads did not match to corresponding protein in BLASTx searches using an E-value of 10^−4^. These abundance estimates are in good agreement with anammox abundance estimates reported for the upper and core regions of the chilean OMZ (Stewart et al., 2012). The total “*Candidatus* Scalindua profunda” gene coverage in station PA2 and PA5 was 13-fold (using 7.8 gigabases; Table 1) and 57-fold (using 8.6 gigabases; Table 1), respectively. The high number of reads matching “*Candidatus* Scalindua profunda” genes in the core of the Arabian sea OMZ (station PA5) made analysis of the diversity in 16S rRNA and core genes of the anammox metabolism possible.

**Table 1:** **Overview Arabian Sea metagenome**.

**Location**	**Station**	**Sequence technology**	**Total reads**	**Average read length**	**Total Mb**	**Reads matching Scalindua^a^**	**Scalindua Mb**	**Genome coverage^b^**	**Percentage Scalindua^c^**
Arabian Sea OMZ	PA2 (170 m)	solid bc3	140622908	50	7031	1320669	66.0	12.9	0.9
Arabian Sea OMZ	PA2 (170 m)	ion torrent	4860488	156	758	31624	2.8	0.5	0.4
Arabian Sea OMZ	PA5 (600 m)	solid bc1	86734835	50	4337	3066081	153.3	30.1	3.5
Arabian Sea OMZ	PA5 (600 m)	solid bc2	79446895	50	3972	2772542	138.6	27.2	3.5
Arabian Sea OMZ	PA5 (600 m)	ion torrent	2588720	131	339	43626	5.7	1.1	1.7

a Mapping settings minimum length coverage 50%, minimum identity 80%.

b Scalindua Mb divided by genome size of Scalindua profunda (5.1 Mb; van de Vossenberg et al., 2012).

c Mb matching Scalindua profunda divided by total Mb in the sample * 100%.

### Presence of core genes of anammox metabolism

On average, the core anammox genes listed in Table 2 had a higher (78.7 fold) coverage than the other genes (57 fold). Most notably hydrazine synthase (*hzs*) and hydrazine dehydrogenase (*hdh*) genes were covered up to no less than 155-fold in station PA5, and 35.9-fold in station PA2. Based on their high coverage in station PA5, *hzs*A and *hdh* were selected to assess diversity of “*Candidatus* Scalindua” at the core of the Arabian sea OMZ (see below).

**Table 2:** **Overview Arabian Sea metagenome data covering selected Scalindua genes**.

**Name**	**Gene**	**Gene length**	**Gene id**	**Combined bc1 bc2 Station PA5 reads/gene**	**St PA5 coverage**	**Station PA2 reads/gene**	**St PA2 coverage**	**PA2/PA5 ratio**	**PA5/PA2 ratio**
Hydrazine synthase BC	*hzs*BC	1930	scal00023	3733	96.7098	1139	29.5078	0.3051	3.2774
Hydrazine synthase A	*hzs*A	2421	scal01318	4525	93.4531	880	18.1743	0.1945	5.1420
Hydrazine dehydrogenase	*hdh*	1587	scal03295	4942	155.7026	1172	36.9250	0.2372	4.2167
Hydroxylamine oxidoreductase	*hao*	1752	scal00421	2474	70.6050	509	14.5263	0.2057	4.8605
Hydroxylamine oxidoreductase	*hao*	1572	scal01317	2508	79.7710	507	16.1260	0.2022	4.9467
Hydroxylamine oxidoreductase	*hao*	1638	scal02110	1503	45.8791	413	12.6068	0.2748	3.6392
Hydroxylamine oxidoreductase	*hao*	1383	scal02116	2006	72.5235	252	9.1106	0.1256	7.9603
Hydroxylamine oxidoreductase	*hao*	1356	scal04133	2605	96.0546	642	23.6726	0.2464	4.0576
Hydroxylamine oxidoreductase	*hao*	1127	scal04164	1622	71.9610	826	36.6460	0.5092	1.9637
Nitrate reductase	*nar*G	3498	scal00863	5366	76.7010	1263	18.0532	0.2354	4.2486
Nitrate reductase	*nar*H	1281	scal00867	2514	98.1265	477	18.6183	0.1897	5.2704
Nitrate reductase	*nar*M	987	scal00868	921	46.6565	284	14.3870	0.3084	3.2430
Nitrate transport protein	*nar*K	1290	scal03007	1490	57.7519	519	20.1163	0.3483	2.8709
Nitrite transport protein	*foc*A	1113	scal00416	1542	69.2722	299	13.4322	0.1939	5.1572
Nitrite transport protein	*foc*A	1086	scal00974	2310	106.3536	283	13.0295	0.1225	8.1625
Nitrite transport protein	*foc*A	1128	scal00975	2958	131.1170	268	11.8794	0.0906	11.0373
cd1 nitrite reductase	*nir*S	1698	scal02098	3489	45.4064	396	11.6608	0.2568	3.8939
Ammonium transport protein	*amt*B	1386	scal00587	2569	92.6768	374	13.4921	0.1456	6.8690
Ammonium transport protein	*amt*B	1332	scal00591	1953	73.3108	459	17.2297	0.2350	4.2549
Ammonium transport protein	*amt*B	1365	scal00594	1736	63.5897	476	17.4359	0.2742	3.6471
Ammonium transport protein	*amt*B	1560	scal00596	1397	44.7756	273	8.7500	0.1954	5.1172
Ammonium transport protein	*amt*B	1482	scal01681	1611	54.3522	591	19.9393	0.3669	2.7259
Ammonium transport protein	*amt*B	1952	scal03708	2091	53.5605	760	19.4672	0.3635	2.7513
CO dehydrogenase/acetyl-CoA synthase alpha subunit	*acs*B	576	scal01234	461	40.0174	39	3.3854	0.0846	11.8205
CO dehydrogenase/acetyl-CoA synthase beta subunit	*acs*A	1226	scal02114	2408	98.2055	880	35.8891	0.3654	2.7364
CO dehydrogenase/acetyl-CoA synthase alpha subunit	*acs*B	576	scal02484	472	40.9722	166	14.4097	0.3517	2.8434
Corrinoid FeS protein	*acs*C	1338	scal02486	1972	73.6921	483	18.0493	0.2449	4.0828
Iron sulfur protein	*isp*	1956	scal02487	2731	69.8108	690	17.6380	0.2527	3.9580
Nickel insertase	*acs*F	774	scal02488	1125	72.6744	364	23.5142	0.3236	3.0907
Corrinoid FeS protein	*acs*D	957	scal02489	1560	81.5047	231	12.0690	0.1481	6.7532
5-methyltetrahydrofolate methyltransferase	*acs*E	786	scal02490	1406	89.4402	302	19.2112	0.2148	4.6556
CO dehydrogenase/acetyl-CoA synthase alpha subunit	*acs*B	781	scal03051	1363	87.2599	287	18.3739	0.2106	4.7491
CO dehydrogenase/acetyl-CoA synthase alpha subunit	*acs*B	680	scal03060	972	71.4706	481	35.3676	0.4949	2.0208
CO dehydrogenase/acetyl-CoA synthase beta subunit	*acs*A	363	scal03758	420	57.8512	154	21.2121	0.3667	2.7273
Methylenetetrahydrofolate reductase	*mfr*	969	scal01287	1763	90.9701	284	14.6543	0.1611	6.2077
Formyltetrahydrofolate synthetase	*fth*S	1715	scal02521	5280	153.9359	1208	35.2187	0.2288	4.3709
Methenyltetrahydrofolate cyclohydrolase	*mch*	912	scal03294	1620	88.8158	305	16.7215	0.1883	5.3115
			Average coverage core genes		78.7279		18.9324		
			Average coverage genome		57.0000		12.9477		

The range of coverage of the octaheme hydroxylamine oxidoreductase *hao* genes was more variable with scal_02116 homolog barely detected at station PA2. The nitrate:nitrite oxidoreductase *nxr*AB and nitrite reductases *nir*S were well covered in both stations. Also genes encoding for nitrite (*foc*A) and ammonium transport (*amt*B) were mapped by a high number of reads (Table 2). The genes encoding for the carbon assimilation via the acetyl-coA pathway were well covered with formyl-tetrahydrofolate synthase at 153-fold coverage standing out. Taken together, the results show that genomic potential for anammox core metabolism is well represented in both the upper and core OMZ of the Arabian Sea, which coincides with the biogeochemical and lipid biomarkers data obtained in previous studies (Jensen et al., 2011; Pitcher et al., 2011).

### Anammox bacteria diversity based on 16S rRNA gene reads

In total, 372 reads derived for the Ion Torrent run of the station PA5 (600 m) sample matched the “*Candidatus* Scalindua profunda” 16S rRNA gene. These reads were submitted to the SINA aligner and compared to the Silva SSU Ref database (http://www.arb-silva.de) resulting in 95 reads assigned to the *Planctomycetes* phylum. 83% of those reads were affiliated to “*Candidatus* Scalindua” of the *Brocadiaceae* family, 2% to the Pla4 lineage, 3% to the Planctomycetes group OM190, 5% to the *Phycisphaeraceae* family and 7% to the *Blastopirellula*, *Gemmata* and *Isosphaera* species cluster according to the SILVA taxonomy. Sequence reads affiliated to the “*Candidatus* Scalindua” cluster were closely related to sequences previously recovered from the Arabian Sea (Woebken et al., 2008).

**Figure 1:**
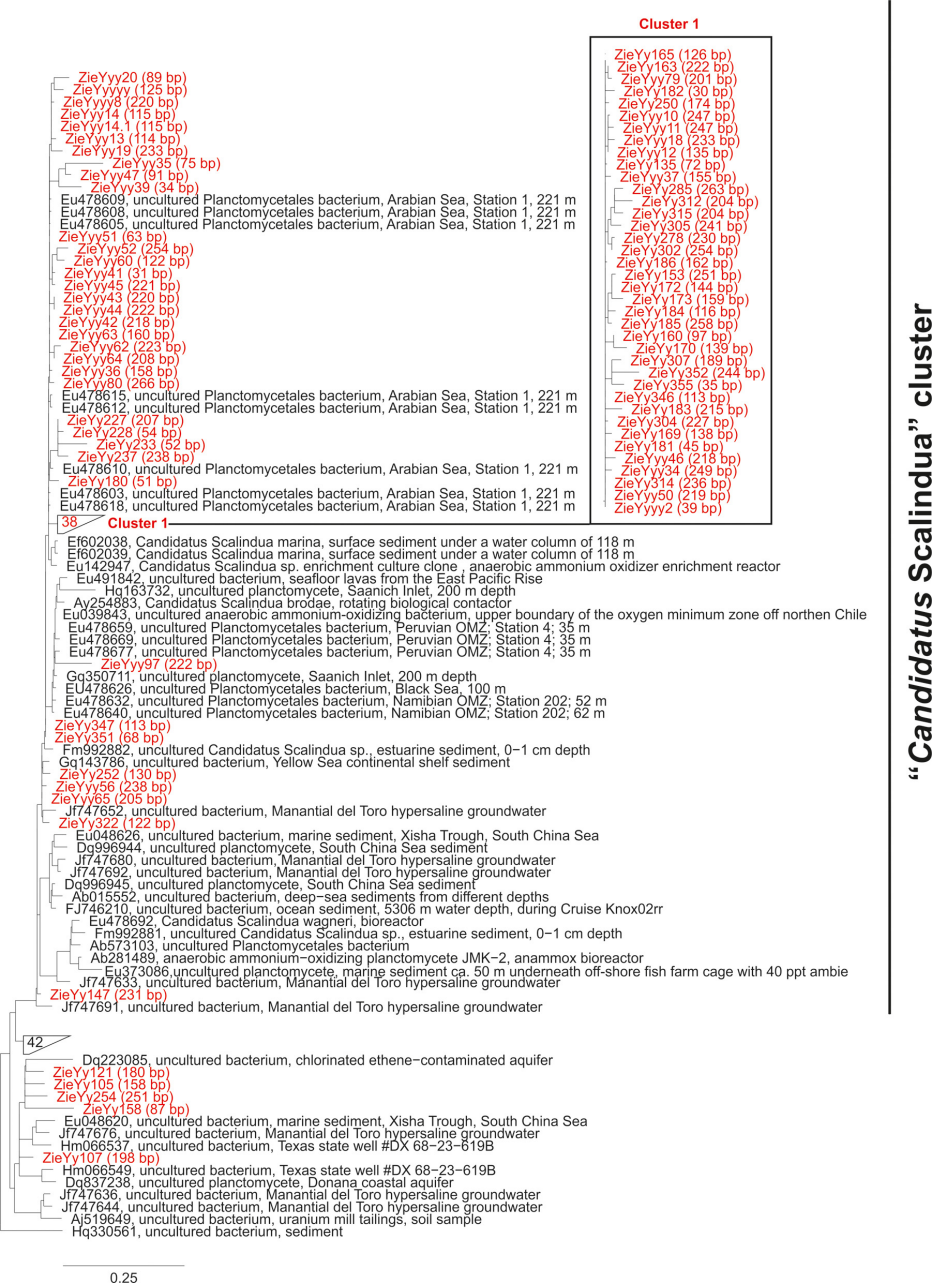
**Maximum likelihood tree of Ion Torrent sequence reads of small subunit rRNA gene sequences obtained from the PA5 (600 m) station of the Arabian Sea that displayed similarity to *Planctomycetes* phylum according to the ARB SILVA database, and close homologs.** Sequence reads derived from our analysis are indicated in red. Sequences previously detected in other OMZ systems are also specified in the tree.

First studies on anammox bacteria diversity based on 16S rRNA gene sequences in suboxic waters, as well as marine and freshwater sediments, concluded that all environmental sequences were closely related to the “*Candidatus* Scalindua” clade and that the diversity was generally low in comparison with other systems, such as wastewater treatment plants (Woebken et al., 2007; Hu et al., 2010). However, another study by Woebken et al. (2008) revealed a significant microdiversity within the “*Candidatus* Scalindua” group by sequence analyses of 16S rRNA genes and the 16S–23S rRNA internal transcribed spacer. In our study, by analyzing the sequence reads obtained from high-throughput sequencing methods, we avoided well-known PCR biases that might result in a misleading interpretation of the anammox bacteria 16S rRNA-based diversity. As seen in Figure 1, most of the reads are closely related to 16S rRNA gene sequences attributed to anammox bacteria previously recovered from the Arabian Sea (Woebken et al., 2008). Some reads are also related to sequences recovered from other OMZ such as Namibia, Peru and Black Sea (Woebken et al., 2008). But in general the diversity detected by 16S rRNA gene reads indicates a very low diversity within the studied anammox population.

### Anammox bacteria diversity based on *hzs*A and *hdh* genes

To further investigate the diversity of the anammox population in the OMZ, the SNV in consensus sequences of the highly covered *hzs*A and *hdh* genes (scal_01318 and scal_03295 respectively) was analyzed. Since the distribution of the SOLiD reads was very non-uniform, only the Ion Torrent reads were taken into account in this analysis. The *hzs*A and *hdh* genes had 33 (1.4%) and 56 (3.7%) variable positions occurring in >20% of the reads respectively. Although Ion torrent reads are prone to homopolymer errors, both coding sequences contained only a single homopolymer stretch longer than four nucleotides (GGGGG in *hzsA* and AAAAAA in *hdh*). Manual inspection confirmed that no variant was called at these sites. Additionally it has been shown elsewhere that, although there is an increase in error rate for homopolymers stretches, it is minimal for stretches up to four nucleotides (Bragg et al., 2013; Ross et al., 2013).

The variable positions were distributed homogeneously over the entire length of the sequence (Table S1). Close inspection of the read mapping suggests that two strains of “*Candidatus* Scalindua” make up the majority of the anammox fraction of our sample. The higher number of SNVs in the *hdh* gene is possibly caused by the presence of two near-identical copies per anammox strain, as has been reported for other anammox bacteria (Strous et al., 2006; Hira et al., 2012).

**Figure 2:**
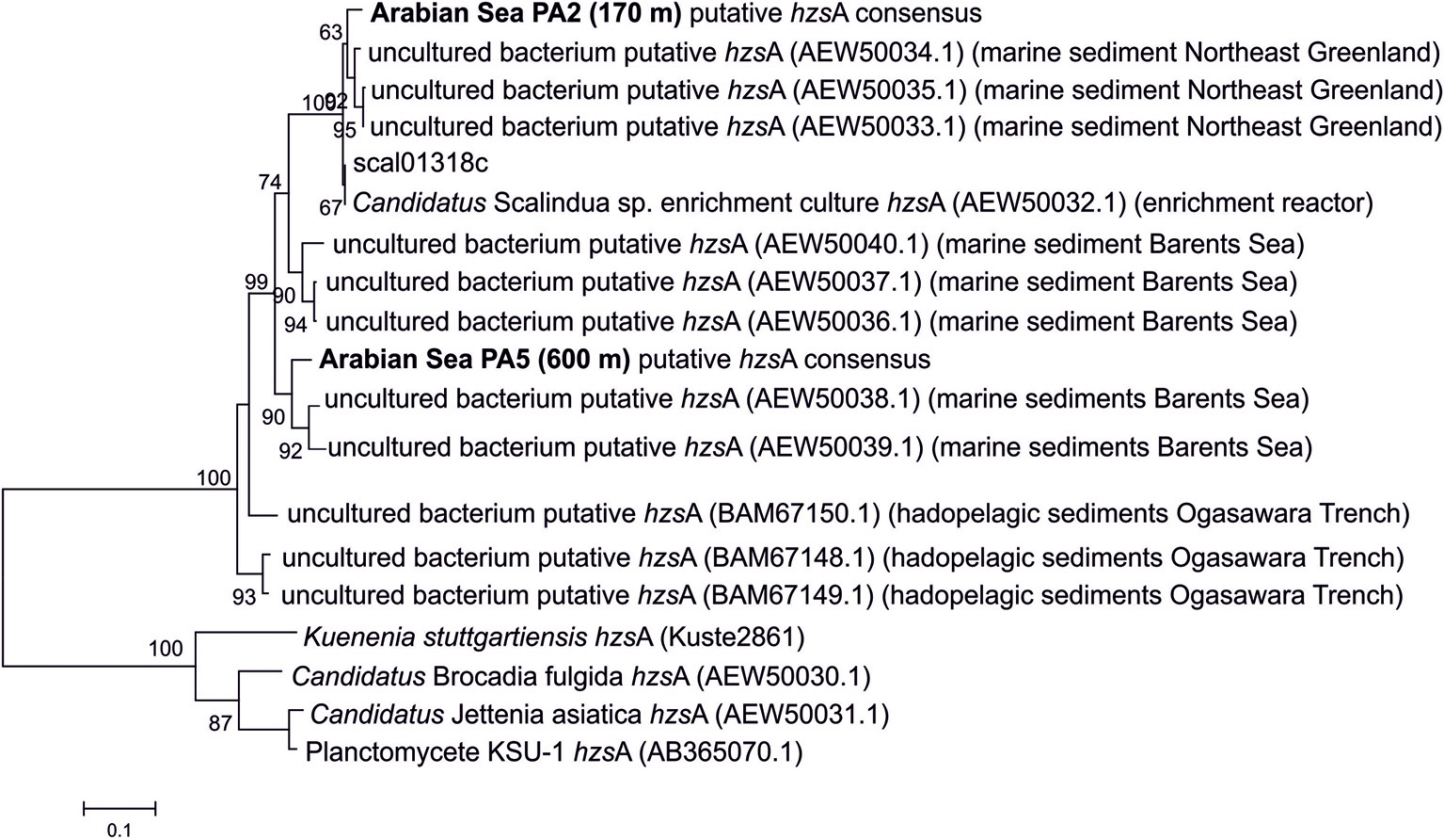
**Maximum likelihood tree of translated *hzs*A sequences based on 426 amino acid positions.** The reference sequence and consensus sequence used in this study are in bold. Branch support was calculated with the approximate likelihood ratio test (aLRT) and indicated on the branches. The scale bar indicates evolutionary distance of 0.1 substitutions per site.

Additionally, the generated consensus sequence of *hzs*A was compared to the *hzs*A of “*Candidatus* Scalindua profunda” and other “*Candidatus* Scalindua” *hzs*A genes previously obtained via PCR amplification (Harhangi et al., 2012; Borin et al., 2013; Nunoura et al., 2013). The final PA5 consensus sequence was 83% identical at nucleotide level to the “*Candidatus* Scalindua profunda” reference and instead clustered with two clones obtained from the Barents Sea (95% identity) (Figure 2). BLASTn against the NCBI nt database confirmed that these Barents Sea sequences are indeed the closest relatives available. *Hzs*A sequences seem to cluster according to environment, but it is worth noting that the number of environments sampled is very low.

Differences in the sequence of the *hzs*A gene in PA2 (170 m) and PA5 (600 m) might indicate the upper and core areas of the OMZ water column are inhabited by different though related anammox populations. These two anammox populations could be segregated based on differences in physicochemical conditions in the Arabian Sea OMZ. Pitcher et al. (2011) reported similar oxygen and nitrite concentrations at 170 and 600 m depth (oxygen, 4.5 and 3.4 μM; nitrite, 0.6 and 0.52 μM, respectively), while ammonia concentrations were 2.5-fold higher at 170 m respect to 600 m depth, suggesting that the anammox population inhabiting the core of the OMZ could be limited in ammonia. The close relation of anammox genes is certainly influenced by the mapping methods used to retrieve the reads that match to the *hzs*A or *hdh* genes. All reads that are more than 20% different are excluded from the analysis. A much higher coverage and a much longer read length, and powerful calculating power would be needed to make better draft assemblies and BLASTx analysis to discover new divergent *hzs*A or *hdh* genes and would also be necessary to discover more divergent anammox species.

## Concluding remarks

NGS technology is a powerful method to obtain genomic information of marine anammox bacteria in OMZ ecosystems. The coverage by SOLiD and Ion Torrent was high enough to analyze the important *hzs*A and *hdh* core anammox genes. Together with 16S rRNA gene phylogenetic analysis, it was shown that the diversity of the marine anammox in the Arabian Sea was lower than previously determined by PCR methods.

### Conflict of interest statement

The Associate Editor declares that despite being affiliated to the same institution as the authors Daan R. Speth and Theo van Alen, the review process was handled objectively and no conflict of interest exists. The other authors declare that the research was conducted in the absence of any commercial or financial relationships that could be construed as a potential conflict of interest.

## Appendix

**Table A1:** **Comparison between mapping and blastx analysis of selected Scalindua genes at station PA5**.

**BLASTX SOLID READS OF PA5**								
			**Blastx**		**Exp = 1,0**	**4583105**		
**Gene**	**Name**	**Mapped reads**	**Hit**	**No blast hit**	**Perc no hit**	**% of total reads**	**Coverage**	**Gene length**
scal_03295	hdh	4942	4137	805	16	0.1078	130.3403	1587
scal_01318	hzsA	4525	3326	1199	26	0.0987	68.6906	2421
scal_00863	narG	5366	3553	1813	34	0.1171	50.7862	3498
scal_02098	nirS	3489	2333	1156	33	0.0761	68.6985	1698
scal_02114	acsA	2408	1554	854	35	0.0525	63.3768	1226
scal_02521	fths	5280	4053	1227	23	0.1152	118.1633	1715
				Average	27.83			
**BLASTX ION TORRENT READS OF PA5**								
					**Exp = 0.0001**	**% of total reads**	**Coverage**	
All genes		22092	22031	61	0.27611805	99.723882		
scal_03295	hdh	80	79	1	1.2500	0.3576	8.7114	1587
scal_01318	hzsA	65	65	0	0.0000	0.2942	4.6985	2421
scal_00863	narG	74	73	1	1.3514	0.3304	3.6521	3498
scal_02098	nirS	49	49	0	0.0000	0.2218	5.0501	1698
scal_02114	acsA	25	25	0	0.0000	0.1132	3.5685	1226
scal_02521	fths	70	70	0	0.0000	0.3169	7.1429	1715
