# Supplementary material for: Shotgun metagenomic data reveals significant abundance but low diversity of “Candidatus Scalindua” marine anammox bacteria in the Arabian Sea oxygen minimum zone
Source: Front Microbiol. 2014 Feb 5;5:31. doi: 10.3389/fmicb.2014.00031 (PMC3913995; doi:10.3389/fmicb.2014.00031)
Supplement: Supplementary file 1 [file DataSheet1.PDF]

Reference id	Reference Position	Type	Length	Reference	Variant	Count	Coverage	Frequency	Forward/reverse balance	Average quality
scal03295c	27	SNV	1	A	G	4	9	44.44	0.5	30.5
scal03295c	39	SNV	1	T	C	3	9	33.33	0.33	31.33
scal03295c	80	SNV	1	A	T	3	12	25	0.33	28.67
scal03295c	90	SNV	1	C	T	6	11	54.55	0.17	30.5
scal03295c	123	SNV	1	T	C	5	15	33.33	0.2	19.8
scal03295c	147	SNV	1	T	C	3	10	30	0.33	29
scal03295c	156	SNV	1	G	A	4	13	30.77	0.5	28.25
scal03295c	168	SNV	1	G	A	4	14	28.57	0.25	26.75
scal03295c	216	SNV	1	A	G	7	16	43.75	0.43	24.71
scal03295c	334	SNV	1	T	G	4	12	33.33	0.5	20
scal03295c	342	SNV	1	A	C	5	14	35.71	0.4	25.8
scal03295c	348	SNV	1	C	T	5	14	35.71	0.4	28
scal03295c	351	SNV	1	C	T	3	15	20	0.33	29
scal03295c	376	SNV	1	A	G	5	13	38.46	0.4	31.4
scal03295c	433	SNV	1	G	A	7	14	50	0.43	24
scal03295c	465	SNV	1	G	T	4	15	26.67	0.5	26
scal03295c	495	SNV	1	T	C	10	24	41.67	0.4	29.3
scal03295c	501	SNV	1	C	T	10	23	43.48	0.3	28.7
scal03295c	513	SNV	1	T	A	11	23	47.83	0.27	30.73
scal03295c	528	SNV	1	C	T	9	20	45	0.22	29.11
scal03295c	534	SNV	1	A	G	11	22	50	0.27	26.91
scal03295c	561	SNV	1	A	G	10	21	47.62	0.3	27.3
scal03295c	609	SNV	1	C	T	5	17	29.41	0.2	25
scal03295c	628	SNV	1	T	G	5	16	31.25	0.2	28.2
scal03295c	629	SNV	1	C	G	5	16	31.25	0.2	28.2
scal03295c	630	SNV	1	A	C	5	16	31.25	0.2	28.2
scal03295c	636	SNV	1	C	T	8	17	47.06	0.5	29.88
scal03295c	640	SNV	1	A	G	6	17	35.29	0.5	29.67
scal03295c	652	SNV	1	G	A	5	13	38.46	0.4	32
scal03295c	681	SNV	1	C	T	6	17	35.29	0.33	26.33
scal03295c	682	SNV	1	C	T	6	16	37.5	0.33	31
scal03295c	687	SNV	1	C	T	5	17	29.41	0.4	30.8
scal03295c	702	SNV	1	T	A	7	16	43.75	0.29	25.57
scal03295c	747	SNV	1	T	A	8	19	42.11	0.38	23.62
scal03295c	768	SNV	1	A	G	6	19	31.58	0.5	25.83
scal03295c	780	SNV	1	T	G	4	19	21.05	0.5	30
scal03295c	819	SNV	1	A	G	3	12	25	0.33	24.67
scal03295c	819	SNV	1	A	T	4	12	33.33	0.5	31
scal03295c	912	SNV	1	C	T	6	12	50	0.17	30.5
scal03295c	981	SNV	1	T	C	3	9	33.33	0.33	21
scal03295c	1044	SNV	1	T	C	4	11	36.36	0.25	25
scal03295c	1074	SNV	1	C	T	3	12	25	0.33	26
scal03295c	1086	SNV	1	T	A	4	12	33.33	0.5	28.75
scal03295c	1101	SNV	1	T	C	4	10	40	0.5	26.25
scal03295c	1152	SNV	1	G	A	5	12	41.67	0.4	32.2
scal03295c	1155	SNV	1	T	C	5	11	45.45	0.4	28.2
scal03295c	1168	SNV	1	A	C	5	13	38.46	0.4	30.2
scal03295c	1169	SNV	1	T	A	5	13	38.46	0.4	30.2
scal03295c	1173	SNV	1	T	C	3	13	23.08	0.33	20.33
scal03295c	1272	SNV	1	T	A	4	11	36.36	0.5	30.25
scal03295c	1275	SNV	1	T	A	3	10	30	0.33	26.67
scal03295c	1284	SNV	1	G	T	4	9	44.44	0.5	28
scal03295c	1422	SNV	1	A	T	5	11	45.45	0.4	29.6
scal03295c	1434	SNV	1	G	A	4	10	40	0.5	27.25
scal03295c	1437	SNV	1	T	C	3	10	30	0.33	33
scal03295c	1449	SNV	1	G	A	3	9	33.33	0.33	33.33
